# Cutting-edge sensation: ankle proprioception during lateral cutting correlates with balance and power in adolescent table tennis players

**DOI:** 10.3389/fbioe.2026.1828725

**Published:** 2026-04-28

**Authors:** Ziwei Cao, Mengde Lyu, Shuhui Wang, Jinglei Huang, Huaibin Qian, Jia Han

**Affiliations:** 1 China Table Tennis College, Shanghai University of Sport, Shanghai, China; 2 School of Biomedical Science and Health, Royal Melbourne Institute of Technology University, Melbourne, VIC, Australia; 3 School of Health and Social Care, Shanghai Urban Construction Vocational College, Shanghai, China; 4 College of Medical Instruments, Shanghai University of Medicine and Health Sciences, Shanghai, China; 5 Graduate School of Shanghai University of Traditional Chinese Medicine, Shanghai, China; 6 Shanghai University of Sport School of Exercise and Health, Shanghai, China

**Keywords:** adolescent, athlete, performance, racket sport, table tennis

## Abstract

**Background:**

Rapid lateral cutting is fundamental to table tennis performance but places high demand on ankle stability and sensorimotor control. Traditional proprioception tests often lack sport-specificity, potentially overlooking the nuanced sensory demands of cutting maneuvers. This study introduces the Ankle Inversion Discrimination Apparatus-Cutting (AIDAC), a novel task-specific tool designed to assess proprioception under movement-specific cutting conditions.

**Objectives:**

To evaluate the reliability and correlation with performance of the AIDAC and to investigate whether ankle proprioception during cutting is linked to key performance indicators, dynamic balance, explosive power, and ankle flexibility, in adolescent table tennis athletes.

**Methods:**

Thirty-two adolescent table tennis players completed the AIDAC, Y-Balance Test (YBT), Weight-Bearing Lunge Test (WBLT), and Single Hop for Distance (SHD). Twenty participants returned for retest. Test–retest reliability was assessed using intraclass correlation coefficients (ICC), coefficient of variation (CV), standard error of measurement (SEM), and minimal detectable change (MDC). Construct validity was examined via correlation analyses between AIDAC scores and performance measures.

**Results:**

The AIDAC demonstrated excellent reliability (ICC = 0.93; CV = 1.91%; SEM = 0.02). Ankle proprioception during cutting was significantly correlated with dynamic balance (YBT: r = 0.52, p = 0.002) and explosive hop distance (SHD: r = 0.47, p = 0.004), but not with static ankle dorsiflexion (WBLT: r = 0.28, p = 0.12).

**Conclusion:**

The AIDAC is a highly reliable tool for assessing sport-specific ankle proprioception. Its strong associations with balance and power highlight the role of cutting-phase sensation in athletic performance and neuromuscular control. This tool offers coaches and clinicians a practical means to screen for proprioceptive acuity.

## Introduction

In the dynamic and high-speed sport of table tennis, an athlete’s ability to move explosively and change direction in response to an opponent’s shot is foundational to performance (Shi et al., 2023). Central to these rapid displacements is the lateral cutting step, a maneuver that demands precise coordination, deceleration, and re-acceleration within milliseconds (Yu et al., 2018). This movement does not occur in isolation; it initiates a kinetic chain that translates lower-limb stability and power into upper-limb stroke quality and accuracy (Shao et al., 2020). However, the same bio-mechanical traits that enable agility also elevate injury risk, particularly to the ankle complex, which must absorb substantial lateral forces while maintaining proprioceptive awareness under time pressure (Lyu et al., 2024).

Proprioception is the sensory feedback governing joint position and movement, which plays a critical role in this context (Han et al., 2016). It enables athletes to perceive ankle inversion angles during cutting, adjust motor responses in real-time, and maintain balance upon landing (Lyu et al., 2024; Han et al., 2021). Deficits in ankle proprioception are closely associated with diminished performance and increased susceptibility to lateral ankle sprains, one of the most common injuries in cutting-dominated sports (Lyu et al., 2024). Traditionally, proprioception has been assessed using generalized protocols such as the Threshold to Detection of Passive Motion or Joint Position Reproduction (Han et al., 2016). While informative, these methods often fail to replicate the sport-specific, weight-bearing, and dynamic nature of movements like cutting, thereby limiting their sport-specific relevance and concurrent validity (Han et al., 2016).

A more functional alternative is the Active Movement Extent Discrimination Assessment (AMEDA), which incorporates natural movement patterns and endpoint detection. Recent advances have led to task-specific AMEDA variants, such as devices for landing (AIDAL), walking (AIDAW), and stair descent, acknowledging that proprioceptive acuity is highly movement-dependent (Han et al., 2022; Wang et al., 2024). Despite this progress, no tool has been designed to evaluate proprioception during the specific kinematic and kinetic demands of a lateral cutting step, a significant gap given the ubiquity of cutting in table tennis and many other court and field sports (Shi et al., 2023; Lyu et al., 2024).

This study introduces the Ankle Inversion Discrimination Apparatus-Cutting (AIDAC), a novel device engineered to assess inversion proprioception during a sport-specific cutting task. This study seeks to determine whether proprioceptive acuity during cutting is functionally linked to key markers of athletic functional performance: dynamic balance, explosive power, and functional ankle mobility. From a theoretical perspective, proprioception underpins neuromuscular control, contributing to postural regulation and dynamic stability, and is therefore closely related to dynamic balance (Wang et al., 2024; Shao et al., 2023). In addition, the ankle-foot complex is the first point of contact with the ground during movement, and thus proprioceptive input may play a mediating role in lower-limb force generation and explosive performance. In contrast, ankle range of motion primarily reflects joint flexibility and structural capacity rather than directly contributing to force mediation during dynamic movements.

Considering previous studies using AMEDA for different tasks, such as the AIDAL (ICC = 0.76), AIDAW (ICC = 0.76), and AMEDA-3D (ICC = 0.82) (Han et al., 2021; Shao et al., 2023; Zhao et al., 2025), we hypothesize that the AIDAC will demonstrate excellent test-retest reliability, and that its measures will correlate significantly with dynamic balance and lower-limb power, but not with passive ankle dorsiflexion range of motion. By establishing the reliability and correlation with performance of the AIDAC, this research aims to provide coaches, clinicians, and sport scientists with a practical, task-specific tool for enhancing performance profiling and functional assessment in athletes for whom cutting is a fundamental component of sport.

## Methods

This cross-sectional study evaluated the reliability and validity of a novel proprioceptive assessment tool during a sport-specific cutting task. The study was approved by ethic committee of local university (approval number: 102772022RT003). All participants and their parents provided written informed consent form before data collection.

### Procedure

Participants attended the testing venue on two separate occasions. During the first visit, the initial AIDAC test was conducted. Upon completion of the AIDAC assessment, participants performed the Y-Balance Test (YBT), Weight-Bearing Lunge Test (WBLT), and Single Hop for Distance Test (SHD). A total of 20 participants returned for a retest after a minimum interval of 7 days following the initial session.

### Participants

A total of 32 adolescent table tennis players (24 males and 8 females; age: 13.03 ± 1.98 years; height: 161.27 ± 12.94 cm; body mass: 47.08 ± 11.86 kg) participated in this study (Table 1). All participants had at least 2 years of professional table tennis training experience and reported no lower-limb injuries within the previous 6 months. Twenty participants (15 males and 5 females; age: 13.53 ± 2.25 years; height: 160.90 ± 13.28 cm; body mass: 46.70 ± 12.21 kg) were randomly selected for a retest at least 7 days after the initial test. No significant differences in age, height, body mass, or initial AIDAC scores were found between the retest group and the full sample (p > 0.05).

**TABLE 1:** Participants characteristics.

Variables	Reliability study	Validity study
Participants, n	20 (15 males and 5 females)	32 (24 males and 8 females)
Age, year	13.53 ± 2.25	13.03 ± 1.98
Height, cm	160.90 ± 13.28	161.27 ± 12.94
Weight, kg	46.70 ± 12.21	47.08 ± 11.86

### Ankle inversion discrimination apparatus-cutting

**FIGURE 1:**
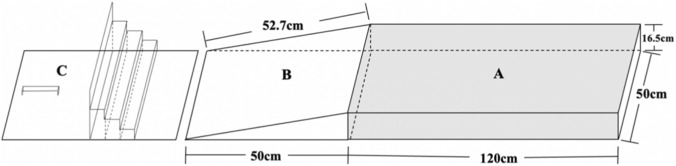
The design of ankle inversion discrimination apparatus-cutting. **(A)** standing platform, **(B)** testing platform, and **(C)** angle-adjustment board.

The development of the AIDAC was based on signal detection theory and the active movement identification method, and consists of three main components (Figure 1): a standing platform (A), a testing platform (B), and an angle-adjustment board (C). The standing platform is used for pre-test standing and for supporting the non-tested foot during side-cutting movements. It measures 120 cm × 50 cm × 16.5 cm. The testing platform, designed to generate various inversion positions, is a wedge-shaped block measuring 50 cm × 50 cm × 16.5 cm, with an inclined surface length of 52.7 cm. During testing, participants are required to perform a complete side-cutting step, placing the tested foot accurately onto the testing platform. The angle-adjustment board (Figure 1C), used in conjunction with the testing platform, provides four different ankle inversion angles of 10°, 12°, 14°, and 16°.

**FIGURE 2:**
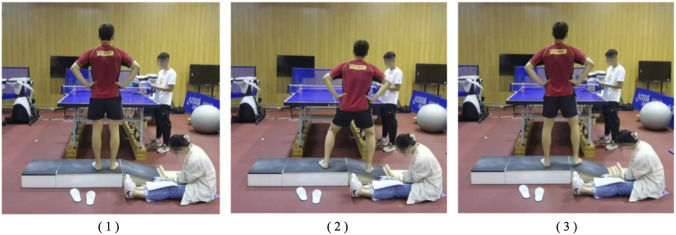
Testing procedure of proprioception. Note: (1) Preparation phase: standing in a ready position with both feet apart; (2) Initiation phase: stepping forward with the test-side foot using a single-step to initiate the cutting movement; (3) Recovery phase: pushing off and returning to the initial position to prepare for the next movement.

Before the formal testing, standardized demonstrations and instructions for the lateral cutting step were delivered to the participants, ensuring that participants performed the movements correctly during the experiment. A complete lateral cutting step consisted of three phases (Figure 2): (1) Preparation phase: standing naturally with both lower limbs apart; (2) Initiation phase: stepping forward with the test-side foot using a single-step; (3) Recovery phase: pushing off and returning to the preparation position to get ready for the next stroke. Participants were instructed to perform the lateral cutting step in a posture consistent with their usual training, and to maintain the same start–end position and step distance as during the learning phase. Once correct execution of the standard movement was confirmed, participants proceeded to the AIDAC familiarization phase. During familiarization, participants stood barefoot on the AIDAC platform with their eyes looking straight ahead. They were given three familiarization trials to experience four different ankle inversion angles and to learn the one-to-one correspondence between each angle and a number (1 = 10°, 2 = 12°, 3 = 14°, 4 = 16°) (Wang et al., 2024; Shao et al., 2023). At this stage, participants were informed of the correspondence but were not required to verbally report it during the familiarization trials. After completing 12 practice trials, participants entered the formal testing phase. The examiner adjusted the inversion angle according to a pre-generated random sequence. Each of the four inversion angles appeared 10 times, for a total of 40 trials. After each stimulus, participants made an absolute judgment of the perceived inversion angle and verbally reported the corresponding number (“1”, “2”, “3”, or “4”) to the examiner. The speed of discrimination was self-selected by the participants, and the entire testing protocol was completed continuously, typically lasting approximately 3 min. Given the very short duration of the test and the low physical exertion required for a single lateral cutting step, no formal rest intervals were deemed necessary, as the risk of physical or mental fatigue was negligible. Furthermore, any potential learning or order effects were rigorously controlled; the four inversion angles were presented according to a pre-generated random sequence created using the RAND function in Microsoft Excel prior to the testing sessions. No feedback regarding the accuracy of the judgment was provided during testing. The inversion angles were randomized using a pre-generated random sequence created prior to testing, ensuring that the order of each angle’s appearance across trials was random (Wang et al., 2024; Shao et al., 2023). The tested limb for each participant was the dominant side, as determined using the Waterloo Footedness Questionnaire (Yang et al., 2018). Test–retest reliability was assessed after a gap of several days, minimizing the influence of short-term practice on reliability outcomes. The area under the receiver operating characteristic curve (AUC) was used to assess ankle proprioception. The total score’s AUC was calculated as the mean of three pairwise AUC values, reflecting the ability to discriminate between adjacent positions in the test. AUC values range from 0.5 (indicating chance-level performance) to 1.0 (indicating perfect discrimination) (Han et al., 2016).

### Y balance test

The YBT was used to assess dynamic balance ability of the lower limbs. The YBT apparatus consisted of one support platform, three movable indicator plates, and three measuring tubes. Participants stood barefoot with the test foot placed on the YBT support platform. The toes of the test foot were aligned with the red line at the front edge of the platform, and the second toe was positioned along the platform’s centerline. Both hands were placed on the hips to eliminate upper limb involvement. During the test, the heel of the test foot was required to remain in contact with the support platform at all times. The non-test foot was used to push the indicator plates in three directions—anterior, posteromedial, and posterolateral—in sequence, reaching as far as possible in each direction. The non-test foot could only touch the ground after completing the pushes in all three directions. The maximum reach distance in each direction was recorded by the examiner. A trial was deemed invalid if any of the following occurred: removal of hands from the hips, lifting of the heel of the test foot, displacement of the test foot, applying body weight on the indicator plate for support, using momentum to push the plate, or placing the non-test foot on the ground before completing all three directions. Each participant completed three formal trials, with a 1-min rest between trials. The average of the three trials was taken as the final YBT composite score (Overmoyer and Reiser, 2015; Zhao et al., 2025). To control for the effects of height and leg length, the reach distances and composite score were normalized to leg length. Composite Score = (Anterior + Posterior Medial + Posterior Lateral)/(3 × Length of Testing Leg) × 100. This composite score was used for statistical analysis.

### Weight-bearing lunge test

The WBLT was used to assess ankle dorsiflexion range of motion. Participants stood facing a wall with both hands placed against it. The test foot was positioned in front and the non-test foot behind, with the midline of the test foot kept perpendicular to the wall. Participants were instructed to touch the wall with the knee of the test limb. Once the knee made contact with the wall, they were asked to gradually increase knee flexion while moving the test foot backward, maintaining contact between the knee and the wall. Throughout the procedure, the heel of the test foot was required to remain in contact with the floor, and no internal or external rotation of the testing limb was allowed. The movement continued until reaching the maximum position where the heel was about to lift off the ground. At this position, the perpendicular distance from the wall to the tip of the first or second toe (whichever was longer) was recorded. Before formal testing, participants were given three practice trials. The formal test consisted of three trials, and the mean value of the three trials was recorded as the WBLT score (Hoch and McKeon, 2011; Powden et al., 2015).

### Single hop for distance test

Participants stood behind the starting line of a 6-m measuring tape in a single-leg stance on the test foot. They were instructed to jump forward as far as possible, landing on the same leg, and to maintain single-leg balance on the test foot for 2 seconds before the non-test foot was allowed to touch the ground. The vertical distance from the starting line to the heel of the landing foot was recorded. A trial was deemed invalid if any of the following occurred: the test foot crossed the starting line prior to take-off, displacement of the landing foot occurred, the single-leg balance time after landing was less than 2 seconds, or the participant touched the ground with their hands to maintain balance. Before formal testing, participants were given three practice trials. The formal test consisted of three trials, each separated by a 1-min rest. The mean distance from the three valid trials was used as the SHD score (Booher et al., 1993).

### Statistical analysis

The normality of data distribution and homogeneity of variances were assessed using the Shapiro–Wilk test and Levene’s test, respectively. Descriptive data were presented as mean ± standard deviation (SD). Reliability of the test measures was evaluated using average-measures intraclass correlation coefficients (ICC) based on a two-way random effects model with absolute agreement, along with 95% confidence intervals (95% CI) and coefficients of variation (CV). ICC values were interpreted according to Koo and Li, where ICC >0.90 indicates excellent reliability, 0.75–0.90 good, 0.50–0.75 moderate, and <0.50 poor (Koo and Li, 2016). The CV was calculated as: CV = (standard deviation of tests 1 and 2/mean of tests 1 and 2) × 100, with values ≤ 10% considered acceptable (Cormac et al., 2008). The standard error of measurement (SEM) and minimal detectable change (MDC) were also calculated to further assess measurement consistency. Between-session effect sizes were calculated using Cohen’s d with 95% CI, where 0.2, 0.5, and 0.8 represent small, medium, and large effects, respectively (Hopkins et al., 2009). Pearson correlation coefficients were used to assess relationships between AUC, Y-Balance Test (YBT), Weight-Bearing Lunge Test (WBLT), and Single Hop for Distance (SHD) performance during the first test session. Correlation magnitudes were interpreted as follows: ≤0.1 (trivial), 0.1–0.3 (small), 0.3–0.5 (moderate), 0.5–0.7 (large), 0.7–0.9 (very large), and 0.9–1.0 (almost perfect) (Hopkins et al., 2009). Statistical significance was set at p < 0.05. All statistical analyses were conducted using IBM SPSS Statistics version 23.0 (IBM Corp., Armonk, NY, USA).

## Results

Table 2 presents the test–retest reliability results for the AIDAC. The ICC for the two testing sessions was 0.93 (95% CI: 0.83–0.97), indicating excellent reliability. No significant differences in proprioceptive acuity were observed between sexes (p = 0.47), and ICC across male and female subgroups ranged from 0.843 to 0.900. The CV was 1.91% (95% CI: 0.03%–2.91%), which was well within the acceptable threshold of <10%. The ES between sessions was −0.24 (95% CI: 0.86 to 0.38), representing a small effect. The SEM was 0.02, indicating that the amount of measurement error was very small.

**TABLE 2:** Reliability, and performance results.

Variables	Reliability study		Validity study
Session 1 AIDAC score	0.802 ± 0.064		0.791 ± 0.068
Session 2 AIDAC score	0.817 ± 0.060		-
ICC (95% CI)	0.93 (0.83, 0.97)		-
CV (95% CI)	1.91 (0.03, 2.91)		-
ES (95% CI)	−0.24 (−0.86, 0.38)		-
SEM	0.02		-
MDC	0.06		-
YBT, %	-	-	103.19 ± 10.39
WBLT, cm	-	-	13.99 ± 2.94
SHD, cm	-	-	149.41 ± 21.37

Abbreviations: AIDAC, Ankle Inversion Discrimination Apparatus-Cutting; ICC, intraclass correlation coefficients; CV, coefficients of variation; ES, effect size; SEM, standard error of measurement; MDC, minimal detectable change; YBT, Y Balance Test; WBLT., Weight-Bearing Lunge Test; SHD, single hop for distance; 95% CI, 95% confidence intervals.

The correlation analysis revealed that ankle inversion proprioception during cutting was significantly associated with YBT (r = 0.52, 95% CI [0.21, 0.77], p = 0.002) and SHD (r = 0.47, 95% CI [0.20, 0.96], p = 0.004), while no significant correlation was found with WBLT (r = 0.28, 95% CI [–0.12, 0.64], p = 0.12).

## Discussion

The primary findings of this study confirm that the AIDAC exhibits excellent measurement reliability and demonstrates significant relationship with athletic performance in adolescent table tennis players. Crucially, the results underscore that proprioceptive acuity is not a general trait but is intrinsically linked to the specific movement in which it is assessed, highlighting the AIDAC’s value as a sport-related assessment.

In the study of reliability, the AIDAC exhibited excellent test–retest reliability in table tennis players, which confirm that the AIDAC provides highly consistent measurements of ankle proprioception performance over time. This level of reliability is consistent with previous studies using AMEDA for different tasks, such as the AIDAL, AIDAW, AMEDA-3D (Han et al., 2021; Shao et al., 2023; Zhao et al., 2025). In addition, the CV was 1.91%, well below the acceptable threshold of <10%, indicating minimal variability in the measurements. The effect size between sessions was −0.24, representing a small effect, suggesting that performance differences across testing sessions were not significant. The SEM was 0.02, indicating a very small measurement error. The stability of these measurements offers a robust basis for functional assessment, training monitoring, and talent identification within this athletic population (Lyu et al., 2024; Han et al., 2015a).

In the study of relationship with sports performance, results showed significant correlations between ankle proprioception scores and both the YBT and SHD performance. These results are consistent with previous studies, suggesting that proprioceptive ability during lateral cutting is closely associated with lower-limb dynamic balance control and explosive strength (Zhao et al., 2025; Han et al., 2015b). The YBT reflects multi-directional dynamic stability and lower-limb coordination, while the SHD primarily indicates joint control during landing. As the ankle–foot complex is the first point of contact with the ground during movement, both abilities critically depend on accurate proprioceptive feedback and neuromuscular control (Han et al., 2015b; Riemann and Lephart, 2013). In contrast, no significant association was observed between the AIDAC and the WBLT. This may be because the WBLT primarily assesses ankle dorsiflexion range of motion and the flexibility of the posterior lower-leg musculature, which are less directly related to the dynamic sensorimotor demands involved in lateral cutting tasks (Hoch and McKeon, 2011; Powden et al., 2015). These findings suggest that the AIDAC may be more reflective of dynamic sensorimotor function rather than static flexibility.

In competitive table tennis, lateral cutting and rapid displacement are frequently used movement patterns during matches (Shao et al., 2020). Athletes need to execute quick movements forward, backward, sideways, and diagonally within very short time frames to respond to the opponent’s varied shots (Shi et al., 2023). This not only requires good ankle proprioception but also highly coordinated neuromuscular control among the lower-limb muscles, as well as sufficient explosive strength and braking ability (Shi et al., 2023). However, these movements also carry a high risk of ankle sprains, anterior talofibular ligament injuries, and repeated lateral cutting may induce significant fatigue (Lyu et al., 2025; Herbaut and Delannoy, 2020; Mohr and Federolf, 2022). Based on these sport-specific characteristics, the AIDAC can serve as a precise tool for assessing proprioceptive ability in table tennis players. By regularly evaluating athletes’ ability to perceive ankle inversion angles during lateral cutting movements, coaches can promptly detect declines in proprioception or deficiencies in neuromuscular control (Lephart et al., 1997). The previous studies highlight the potential value of proprioceptive ability in talent identification and athlete selection, providing coaches and sports science teams with a scientific basis for identifying athletes with superior neuromuscular control at early stages of development (Shi et al., 2023; Han et al., 2015a). In addition, cutting and lateral movements also commonly occur in other sports, such as basketball and badminton (Lyu et al., 2025; Herbaut and Delannoy, 2020; Taylor et al., 2017). While applied in table tennis, the principle of movement-specific assessment and the design of the AIDAC have direct translatability. Sports such as basketball, soccer, badminton, and tennis all feature frequent, high-intensity cutting maneuvers where ankle proprioception is paramount for performance and safety. The AIDAC, therefore, represents not just a new device, but a shift in assessment towards greater movement specificity for court and field sports.

This study has several limitations. The sample was limited to adolescent table tennis players, and the unbalanced sex ratio may limit generalizability. Future studies should include adults, other sports, and consider balanced sex distributions or subgroup analyses. Although an exploratory analysis revealed no significant differences in AIDAC scores between sexes, this finding should be interpreted with caution. Given the small number of female participants, this comparison is likely underpowered, and the absence of statistical significance does not equate to equivalence or full generalizability across sexes. While links to general performance were established, future research should examine associations with sport-specific outcomes, such as on-court footwork speed, stroke accuracy, or injury incidence. Another limitation is that individual differences in maturation status were not considered and may have influenced proprioception and performance outcomes. Although the AIDAC incorporates key kinematics of a lateral cutting step, testing was conducted in a controlled environment without a racket or ball, limiting task-specific conditions. However, this design improves measurement reliability by isolating proprioceptive processing. Future studies may include more game-like elements and investigate the AIDAC’s responsiveness to interventions to further support its use as a monitoring tool. Importantly, the cross-sectional design of this study only permits conclusions regarding test-retest reliability and concurrent validity. It does not establish the predictive validity of the AIDAC for injury risk or its longitudinal effectiveness, which requires future prospective cohort studies.

## Conclusion

The AIDAC is a reliable proprioception testing device that can be used to specifically assess proprioception during cutting movements in table tennis athletes. Future applications should extend to athletes in other sports.

## Data Availability

The raw data supporting the conclusions of this article will be made available by the authors, without undue reservation.
